# Feasibility Study of Suitable Surface Treatments for 3D-Printed Parts to Increase Abrasion Resistance Stability

**DOI:** 10.3390/polym18060703

**Published:** 2026-03-13

**Authors:** Dominik Fink, Zdenek Chval, Karel Raz

**Affiliations:** Faculty of Mechanical Engineering, Regional Technological Institute, University of West Bohemia, Univerzitni 2732/8, 301 00 Plzen, Czech Republic; zdchval@fst.zcu.cz (Z.C.); kraz@fst.zcu.cz (K.R.)

**Keywords:** additive manufacturing, Multi-Jet Fusion, PA12GB, surface treatments, surface coatings, chemical vapor smoothing, abrasion resistance, tribological properties, surface roughness, Shore D hardness

## Abstract

Additive manufacturing technologies such as Multi-Jet Fusion (MJF) enable the production of polymer parts with relatively isotropic mechanical properties; however, their surface condition often limits direct functional application. This study investigates the feasibility of selected surface treatments applied to PA12GB (glass bead-filled PA12) parts manufactured by MJF, with the aim of improving abrasion resistance and temperature-related performance through the modification of surface properties. Five surface treatments were evaluated: base coating (BC), acrylic coating (AC), chemical vapor smoothing (PostPro3D), glasscoat (epoxy-based SiO_2_ system), and a ceramic-filled 2K epoxy coating. Untreated samples served as a reference. Surface layer thickness, roughness (ISO 21920-2:2021), coefficient of friction (ASTM G99-23), and Shore D hardness (ASTM D2240-15R21) were measured. The results showed significant differences among treatments. Glasscoat and ceramic coatings formed the thickest and hardest layers (≈265 μm and ≈409 μm; Shore D ≈ 84) but exhibited substantially increased friction coefficients. Vapor smoothing and BC produced thinner layers with properties comparable to untreated samples. Acrylic coating reduced surface roughness while moderately increasing hardness. The findings demonstrate that surface treatments substantially alter the tribological and mechanical surface behavior of MJF-printed PA12GB parts. The suitability of a given treatment strongly depends on the intended functional requirements, particularly with respect to friction and surface hardness.

## 1. Introduction

Additive manufacturing (AM) has become a widely adopted production technology for functional polymer components due to its ability to produce geometrically complex parts with high dimensional flexibility and reduced tooling requirements [1,2,3]. Surface quality and dimensional accuracy remain key parameters influencing the functional applicability of AM parts [1,2].

Among polymer powders used in powder bed fusion technologies, polyamide 12 (PA12) is one of the most established materials due to its balanced mechanical properties and process stability [4]. Multi-Jet Fusion (MJF) represents an advanced powder bed fusion technology for polymer processing, offering improved productivity and relatively isotropic mechanical properties compared to extrusion-based processes [5,6]. The addition of glass beads to PA12 (PA12GB) further modifies its stiffness and thermal behavior, although it may also influence its mechanical response and surface morphology [7,8].

Despite favorable bulk properties, MJF-produced parts typically exhibit relatively high surface roughness and partial surface porosity resulting from the layer-wise sintering of powder particles [9,10]. The presence of partially fused particles and surface asperities can negatively affect tribological performance and long-term reliability [6,9]. For applications involving sliding contact or mechanical loading, surface modification is therefore often required.

A wide range of post-processing technologies have been investigated to improve the surface characteristics of additively manufactured polymers [11,12]. Mechanical finishing, plasma treatment, and chemical surface modification have demonstrated the potential to reduce surface roughness or enhance adhesion properties [13,14,15]. Thermal post-treatments can further alter crystallinity and mechanical performance [16,17].

From a functional perspective, tribological behavior is particularly important. Previous studies have shown that wear resistance and friction behavior of polyamide-based materials are significantly influenced by filler content and surface condition [18,19]. However, although numerous studies focus on individual post-processing techniques, a direct experimental comparison of several coating-based and chemical surface treatments applied to MJF-produced PA12GB parts under identical testing conditions remains limited.

From a materials science perspective, the performance of coated polymer systems is primarily governed by interfacial adhesion mechanisms between the substrate and the applied surface layer. Adhesion in polymer–coating systems generally arises from a combination of mechanical interlocking and chemical bonding mechanisms [20,21,22]. Mechanical interlocking is particularly relevant for additively manufactured components, where inherent surface roughness and open porosity may enable the physical anchoring of low-viscosity coatings within surface asperities and pores. In contrast, chemical bonding mechanisms depend on interfacial compatibility, surface energy, and the potential formation of covalent or secondary intermolecular bonds between the coating matrix and the polymer substrate [23].

In the case of MJF-produced PA12GB, the presence of partially fused powder particles and glass bead fillers creates a heterogeneous surface morphology and a multiphase interface, which may significantly influence wetting behavior, penetration depth of coatings, and stress transfer across the interface. The balance between mechanical anchoring and physicochemical adhesion therefore plays a crucial role in determining coating stability, frictional response, and wear resistance [20,21,22,23].

Although individual post-processing strategies have been investigated in previous studies, a systematic comparison of surface treatments with fundamentally different adhesion mechanisms applied to MJF-printed PA12GB under identical tribological testing conditions remains limited. Understanding how interfacial bonding mechanisms influence macroscopic friction and hardness is essential for the application-oriented optimization of additively manufactured functional components.

The aim of the present study is therefore to evaluate selected surface treatments applied to MJF-printed PA12GB components and to assess their influence on surface roughness, layer thickness, friction coefficient, and hardness. Surface roughness parameters were evaluated according to the ISO 21920-2:2021 standard for surface texture characterization [24]. Tribological testing was performed using the pin-on-disk method according to ASTM G99-23 [25], and hardness was measured according to ASTM D2240-15R21 [26]. The goal is to determine which treatment strategy provides the most suitable balance between abrasion resistance and surface mechanical performance. The evaluated surface treatments therefore represent systems with fundamentally different adhesion mechanisms and stiffness characteristics, enabling a mechanistic interpretation of their tribological performance. In the present feasibility-oriented approach, coating performance is assessed primarily through functional tribological response rather than direct adhesion strength testing.

Although several studies have investigated surface finishing techniques for additively manufactured polymer components, a systematic comparison of commercially available coating systems and post-processing technologies for improving tribological performance remains limited. In particular, limited attention has been given to the combined evaluation of coating thickness, surface roughness, hardness, and friction behavior in glass-filled PA12 components produced by Multi-Jet Fusion.

Therefore, the aim of this study is to evaluate the feasibility of selected surface treatments for improving the abrasion resistance and tribological properties of MJF-produced PA12GB components. The work focuses on a comparative analysis of several commercially available coating systems and vapor smoothing technology, with emphasis on their influence on surface morphology, friction behavior, and wear resistance.

## 2. Materials and Methods

An integral part of 3D prints or additive manufacturing (AM) intended for operational use is post-processing, which mainly consists of modifying the surface of the prints [13,14]. The aim is to create a layer on the surface of the prints that improves mechanical (friction), thermal, chemical, or abrasive properties, or eliminates the negative impact of the inherently rough 3D-printed surface [9,10,11,12].

With regard to the MJF technology under consideration, which produces prints with more isotropic material properties than FDM technology [5,6], nylon-based prints reinforced with glass beads (PA12GB) were used in this study. The mechanical behavior of glass bead-filled PA12 differs from neat PA12 and depends on both the manufacturing process and reinforcement content [7,8,27]. Given the powder bed production mechanism, post-processing of the print surface is necessary because the prints are created by layering powdered material and sintering it in a closed container. The resulting surface morphology is often comparable to sand-cast components due to the presence of partially fused particles [9,25,26,27,28,29,30,31].

**Figure 1:**
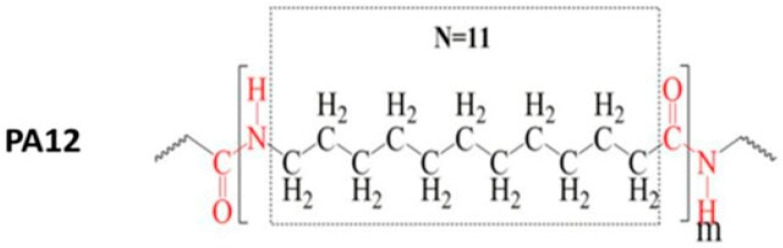
Chemical structure of polyamide 12 (PA12) [31].

The base material used in this study was polyamide 12 reinforced with glass microspheres (PA12GB). Polyamide 12 is a semicrystalline thermoplastic polymer belonging to the polyamide family. Its molecular structure consists of repeating amide groups (–CONH–) connected by aliphatic hydrocarbon chains, as illustrated in Figure 1. This structure provides a combination of good mechanical strength, chemical resistance, and relatively low moisture absorption compared to other polyamides.

The presence of polar amide groups in the polymer chain also plays an important role in surface interactions and adhesion behavior with applied coatings.

For this reason, in industrial practice, the surface is typically cleaned of residual powder and surface roughness is reduced using abrasive blasting, glass bead blasting, or other conventional finishing techniques [9,25,32]. Less commonly, vibratory or chemical polishing of surfaces is also used [18,33].

This operation is usually followed by further surface treatment, such as dyeing, which utilizes the porous surface layer characteristic of powder bed manufactured polymers [11,26]. However, these processes primarily improve aesthetic appearance and surface uniformity but do not significantly enhance tribological performance, hardness, or temperature stability [12,23,34].

A porous surface is advantageous for dye absorption but unsuitable for sliding or mechanically loaded applications. Since the tribological behavior of polyamide materials is strongly influenced by surface condition and fillers [32,33], several experiments were conducted to identify suitable surface treatments that could improve the functional performance of PA12GB prints. Emphasis was placed on friction properties and resistance to temperature-related effects.

Improvements in friction behavior can be achieved either by modifying surface roughness or by applying a protective coating layer [18,19,24,25,26,27,28]. Protective layers may additionally increase surface hardness and thermal stability [22,35,36]. Based on these assumptions, several surface treatments were selected for experimental evaluation.

Twenty test specimens in the shape of disks (70 mm diameter) were produced. Four specimens were assigned to each surface treatment group. The selected surface treatments were subsequently evaluated in terms of surface roughness (ISO 21920-2) [24], coefficient of friction using the pin-on-disk method (ASTM G99) [25], and surface hardness (ASTM D2240) [26]. The thickness of the applied surface layers was determined using optical microscopy (Olympus, Prague, Czech Republic). The individual treatments were compared to determine their suitability for improving abrasion resistance and surface mechanical performance.

The present experimental program focused on surface roughness, layer thickness, friction coefficient, and hardness measurements. Standardized adhesion strength tests such as cross-cut (ISO 2409 [37]) or pull-off tests (ISO 4624 [38]) were not performed. The evaluation of coating performance was therefore based on functional tribological response rather than direct interfacial strength quantification.

**Figure 2:**
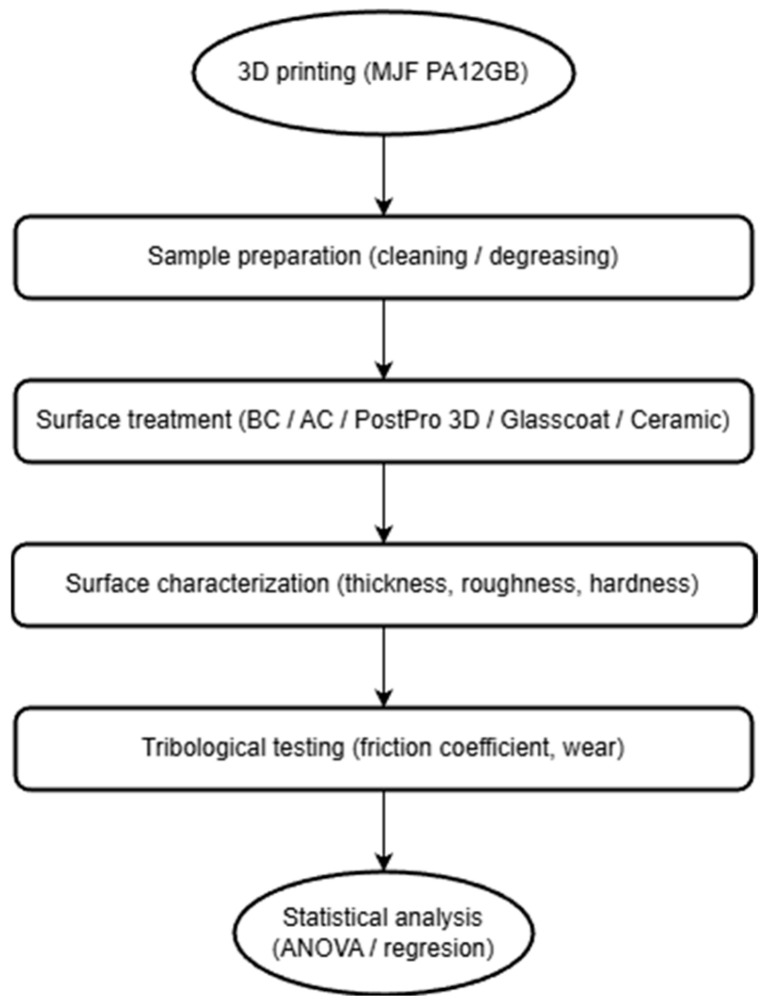
Experimental workflow of the study.

The overall experimental workflow of the study is summarized in Figure 2.

### 2.1. Sample Preparation

Test specimens were manufactured using Multi-Jet Fusion (MJF) technology from PA12GB material reinforced with glass microspheres. The specimens were produced using standard printing parameters recommended by the manufacturer.

A total of 24 specimens were fabricated and subsequently divided into experimental groups according to the applied surface treatment. Prior to the coating application, all samples were cleaned and degreased to ensure proper adhesion of the applied surface treatments.

The specimens were then subjected to different post-processing procedures, including vapor smoothing (PostPro3D) and several commercially available coating systems. Each treatment group consisted of multiple specimens used for thickness, roughness, hardness, and tribological measurements.

### 2.2. Samples Without Surface Treatment

**Figure 3:**
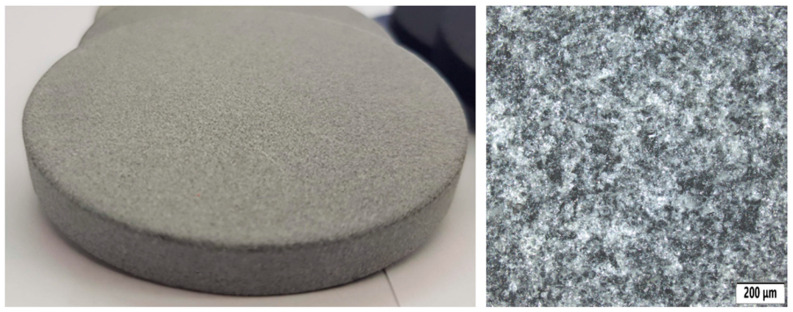
Sample without surface treatment (**left**) and its microstructure (**right**).

For an absolute comparison with the basic state of the tested material for 3D printing, a number of samples were created that were not treated with any surface finish. Their surfaces were only cleaned as a standard part of the post-processing operation for MJF technology. A photo of the sample can be seen in Figure 3.

The image shows that the surface is relatively rough, which is also apparent to the touch. The surface also appears to be relatively porous.

### 2.3. Samples with Base Coating (BC) Surface Treatment—Base Layer with Filler

Base coating (BC) represents a primer-type surface layer containing filler particles intended to improve adhesion between the substrate and subsequent coatings. In polymer systems, such primer layers may enhance interfacial bonding by penetrating surface asperities and pores, thereby promoting mechanical interlocking.

In the present study, the BC layer was applied to evaluate its ability to modify surface morphology and influence tribological response. Due to its filler content and intermediate stiffness, the coating is expected to alter surface roughness while maintaining relatively similar hardness to the polymer substrate.

**Figure 4:**
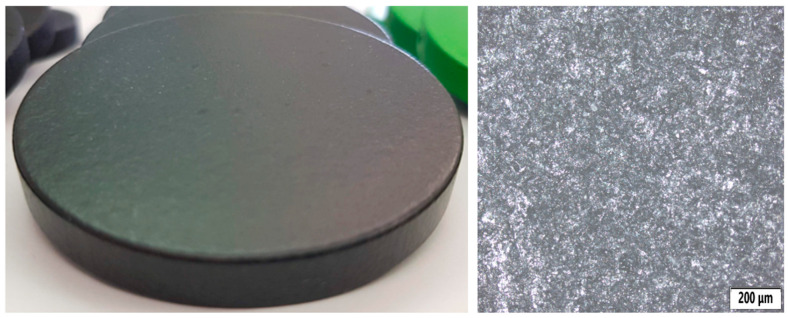
Sample with base coating (BC) surface treatment (**left**) and its microstructure (**right**).

This surface treatment is usually applied to the substrate—such as metal, plastic, or wood. A photo of the sample can be seen in Figure 4. Its main properties are:

- Improving the adhesion of subsequent layers (e.g., paint, varnish);
- Protecting the surface against corrosion, oxidation, or other damage;
- Smoothing out unevenness and creating a smooth surface.

Base coating can be applied, for example, as an anti-corrosion coating on metal parts or as a primer in painting processes. Typical forms include coating, spraying, or dipping. It is commonly used in the automotive, engineering, and construction industries.

### 2.4. Samples with Acrylic Coating (AC)—Sprayed with Acrylic Paint

The acrylic coating (AC) is based on crosslinked acrylic polymers forming a continuous protective film on the substrate surface. Acrylic systems are characterized by relatively low modulus compared to ceramic-filled coatings and exhibit good flexibility and UV stability.

**Figure 5:**
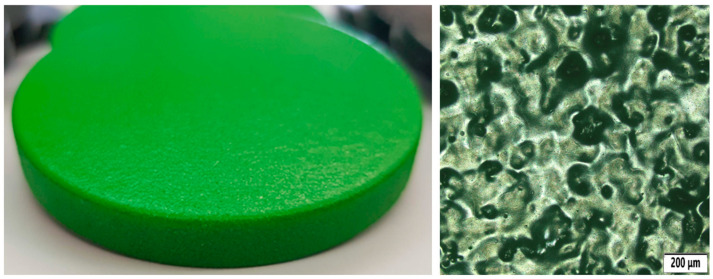
Sample with acrylic coating (AC) surface treatment (**left**) and its microstructure (**right**).

From a materials perspective, the AC layer represents a compliant polymeric coating with potential to reduce surface roughness by filling surface irregularities. Its influence on friction behavior may therefore be governed primarily by topographical modification rather than substantial changes in interfacial shear strength. A photo of the sample can be seen in Figure 5. It is characterized by:

- High resistance to UV radiation, weathering, and chemicals;
- Fast drying and easy application (with a brush, roller, or spray);
- Good elasticity, which prevents cracking during material expansion;
- Versatility for use on various surfaces—concrete, metal, plastic, and wood.

Acrylic coating is often used in construction (e.g., facades and roofs) or in the automotive and furniture industries as a protective and colored coating. It is available in various shades and glosses.

### 2.5. Samples Vaporized Using the PostPro3D Method

The PostPro3D vapor smoothing process is a chemical surface modification technique based on controlled solvent vapor exposure. The solvent partially dissolves and reflows the outer polymer layer, resulting in reduced surface asperities and the partial sealing of open porosity.

Unlike applied coatings, vapor smoothing does not introduce a distinct foreign material layer but modifies the near-surface region of the polymer itself. The adhesion mechanism is therefore intrinsic, and changes in tribological performance are expected to result primarily from the altered surface topography and modified near-surface microstructure.

**Figure 6:**
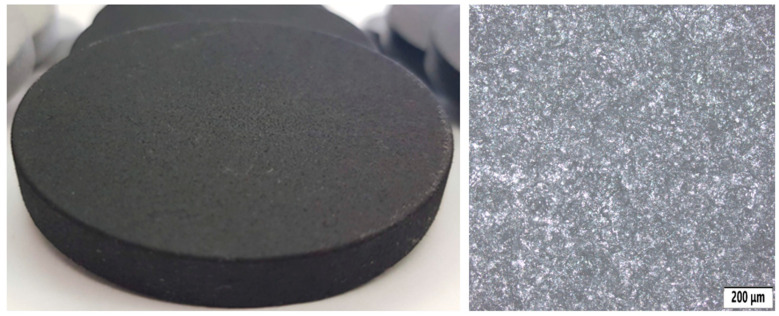
Sample vaporized using the PostPro3D method (**left**) and its microstructure (**right**).

The technology was developed by AMT (Additive Manufacturing Technologies) and is primarily intended for polymer parts manufactured using methods such as SLS, MJF, or FDM. A photo of the sample can be seen in Figure 6. Its main properties are:

- Chemical vapor (special solvent) slightly etches the surface and smooths the print layers.
- Creates a smooth, glossy, and water-resistant surface.
- Does not affect the dimensional accuracy or mechanical properties of parts.
- Fully automated—no manual intervention is required.

A significant advantage is the repeatable quality and speed of processing, even for a larger series of parts. That is why it is used in the industrial production of functional parts, design prototypes, and end products, for example in the automotive, healthcare, and consumer electronics industries.

### 2.6. Samples with Glasscoat Surface Treatment—Coated with Two-Component Epoxy Resin

The glasscoat treatment consists of a two-component epoxy system filled with silicon dioxide (SiO_2_) nanoparticles, forming a rigid crosslinked composite layer. Such systems typically exhibit high hardness and low elastic compliance due to their inorganic filler content and dense polymer network.

From an interfacial perspective, adhesion may arise from a combination of mechanical interlocking within surface pores and physicochemical bonding between the epoxy matrix and the polyamide substrate. The high stiffness contrast between the coating and substrate may significantly influence stress distribution under sliding contact.

**Figure 7:**
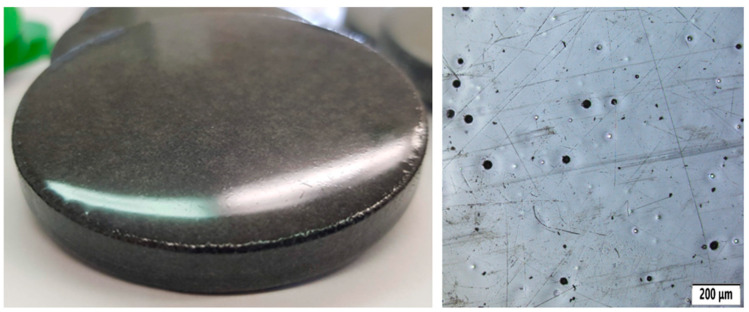
Sample with glasscoat surface treatment (**left**) and its microstructure (**right**).

When combined with epoxy, it forms a two-component composite mixture that creates a hard, thin, and transparent glass-like film on the surface. A photo of the sample can be seen in Figure 7. Its main properties are:

- High hardness and resistance to scratches, chemicals, and UV radiation.
- Hydrophobic effect—repels water and dirt and facilitates cleaning.
- Long service life—lasts significantly longer than conventional waxes or sealants.
- Enhances the shine and depth of the surface color.

Glasscoat is often used in the automotive industry for details (on car bodies, glass, wheels), but also on ceramics, glass, plastics, or painted surfaces in industry and construction.

### 2.7. Samples with a Ceramic Coating Based on 2K Epoxy (Loctite 7227)

The ceramic-filled two-component epoxy coating (e.g., Loctite 7227) represents a highly reinforced composite surface layer containing hard ceramic particles dispersed within a crosslinked epoxy matrix. Such systems are designed to provide enhanced abrasion resistance through increased surface hardness and load-bearing capacity.

The presence of rigid ceramic fillers is expected to increase the plowing component of friction during sliding contact against a steel counter-body. Adhesion to the polymer substrate may depend strongly on wetting behavior and penetration into surface porosity, while internal stress concentration at the coating–substrate interface may influence durability.

**Figure 8:**
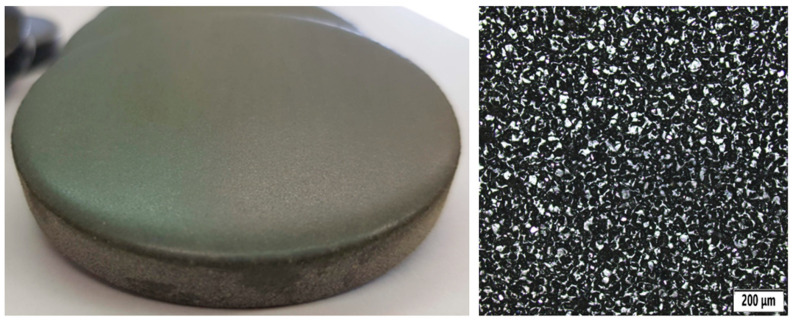
Sample with a ceramic coating based on 2K epoxy (Loctite 7227) surface treatment (**left**) and its microstructure (**right**).

This surface system filled with ceramic particles was designed to protect metal surfaces from mechanical wear, abrasion, and corrosion. A photo of the sample can be seen in Figure 8. The main properties of this coating are:

- High resistance to abrasion, erosion, and chemicals;
- Excellent adhesion to various metal substrates;
- Thermal and chemical stability;
- Easy application with a brush or trowel.

This type of coating creates a hard and smooth ceramic layer that prevents deposits and increases the efficiency of fluid flow. It is therefore used to protect pumps, valves, pipes, tanks, and other industrial equipment exposed to wear and tear, or to restore damaged surfaces and extend their service life.

Due to manual application, a certain variability in coating thickness was observed (see Table 1). Although this approach reflects common industrial practice, it inherently reduces thickness uniformity. The potential influence of thickness variation on tribological performance is therefore considered in the interpretation of the results.

All reported mean values are presented together with the sample standard deviation (SD) and variance to ensure statistical transparency and to assess repeatability. The variance was calculated as the square of the sample standard deviation. The number of measurements for each parameter is specified in the corresponding tables. No data filtering or outlier removal was applied.

Statistical differences between the experimental groups were evaluated using one-way ANOVA (α = 0.05). When statistically significant differences were detected, pairwise comparisons were performed using two-sample *t*-tests assuming unequal variances.

## 3. Results

Several measurements and tests were performed on all the samples to verify the effect of the sample’s surface treatment on increasing abrasion resistance stability.

Statistical significance between the investigated surface treatments was evaluated using one-way ANOVA (α = 0.05). The analysis revealed statistically significant differences between the groups for all evaluated parameters, including coating thickness, hardness, friction coefficient, and surface roughness (*p* < 0.001).

### 3.1. Thickness Measurement of Surface Treatment Layers

The coating thickness measurement required destructive sample preparation. Therefore, after the completion of tribological testing, one representative specimen from each surface treatment group was sectioned for cross-sectional analysis.

The thickness of the surface layer was measured for all samples with surface treatments. The measurement was performed using an Olympus BX61 microscope (Olympus, Prague, Czech republic) (upright optical (light) microscope equipped with a 10× objective (NA 0.25)).

**Figure 9:**
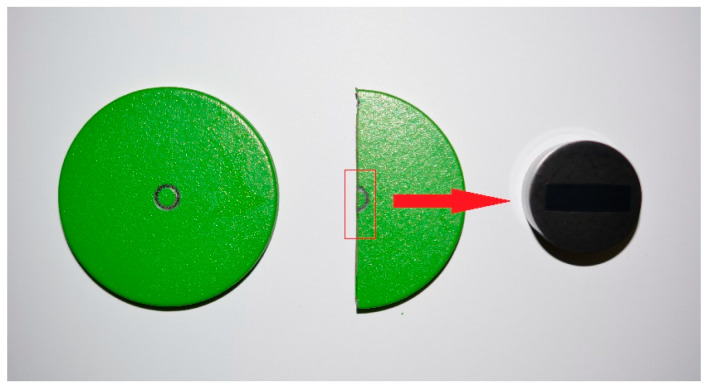
Preparation of samples for measuring the thickness of the surface layer.

To perform the measurement, it was therefore necessary to prepare the sample as shown in Figure 9:

- A cut was made through the center of the disk (sample);
- A 20 mm long sample was extracted;
- A cross-section perpendicular to the surface with a surface layer was prepared;
- 15 measurements/samples were taken (excluding traces from the friction test).

**Figure 10:**
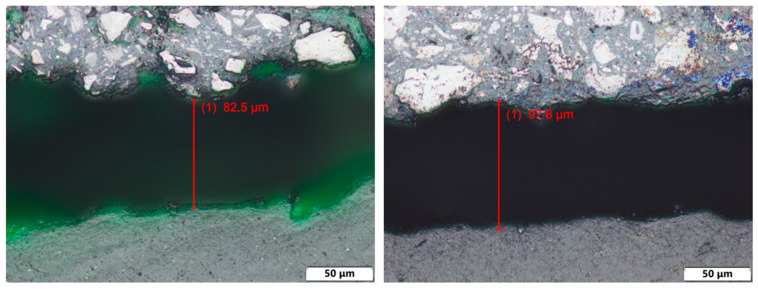
Thickness of surface layer measurement examples of PA12-AC (**left**) and PA12-BC (**right**).

**Figure 11:**
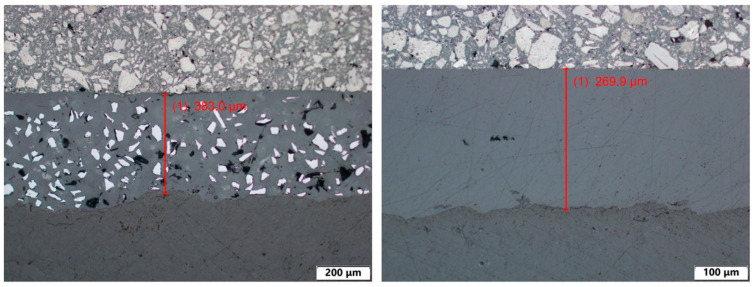
Thickness of surface layer measurement examples of PA12-ceramic (**left**) and PA12-glasscoat (**right**).

**Figure 12:**
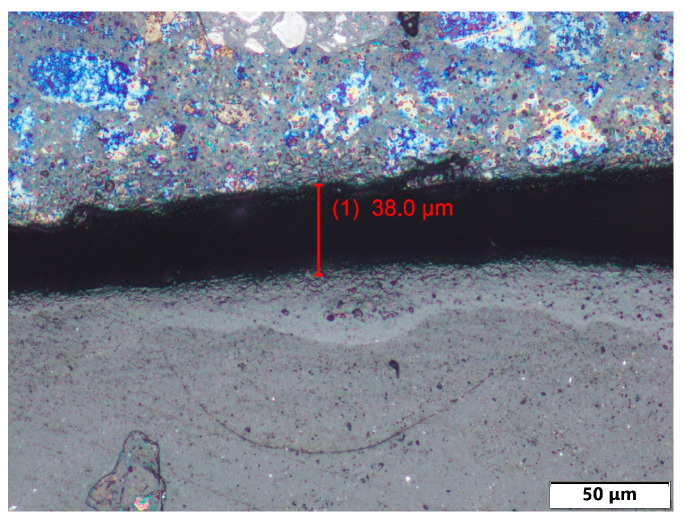
Thickness of surface layer measurement examples of PA12-PostPro3D.

Local thickness measurements were performed along the coating cross-section at different positions, excluding the wear track area. The reported average thickness values therefore represent the mean of fifteen local measurements obtained from one representative specimen per treatment group. The results of the layer thickness measurement are shown in Table 1. Examples of thickness measurement results can be seen in Figure 10, Figure 11 and Figure 12.

The measurement results show that surface coating technologies result in significant differences in surface layer thicknesses. The thinnest surface layers were achieved with PostPro3D vaporization (44.5 μm), followed by almost double that thickness with the BC layer (93.1 μm). Next was the AC surface layer (106.4 μm). The surface treatments with significantly greater layer thicknesses are glasscoat (264.7 μm) and ceramic spraying (409.1 μm).

The calculated statistical values indicate that the thick ceramic and glasscoat systems exhibited relatively low relative thickness variability (6.4–7.4%), whereas thinner layers such as PostPro3D and acrylic coating showed higher local variability (~24%). This suggests that manual application did not significantly compromise the thickness uniformity of the ceramic system.

The differences in coating thickness between individual surface treatments were statistically significant according to the one-way ANOVA analysis (*p* < 0.001).

### 3.2. Measurement of Surface Roughness

For each surface treatment, surface roughness was measured using an Olympus LEXT OLS 5000 (Olympus, Prague, Czech republic) confocal laser scanning microscope and surface roughness parameters were evaluated according to ISO 21920-2:2021.

The measurement was performed in two perpendicular directions—10 fields of view by 10 fields of view. The imaging was performed using a 5× magnification lens. The sample was aligned using three points to reduce noise peaks. Furthermore, a coefficient of λc = 0.8 mm = 800 μm was automatically assigned.

**Figure 13:**
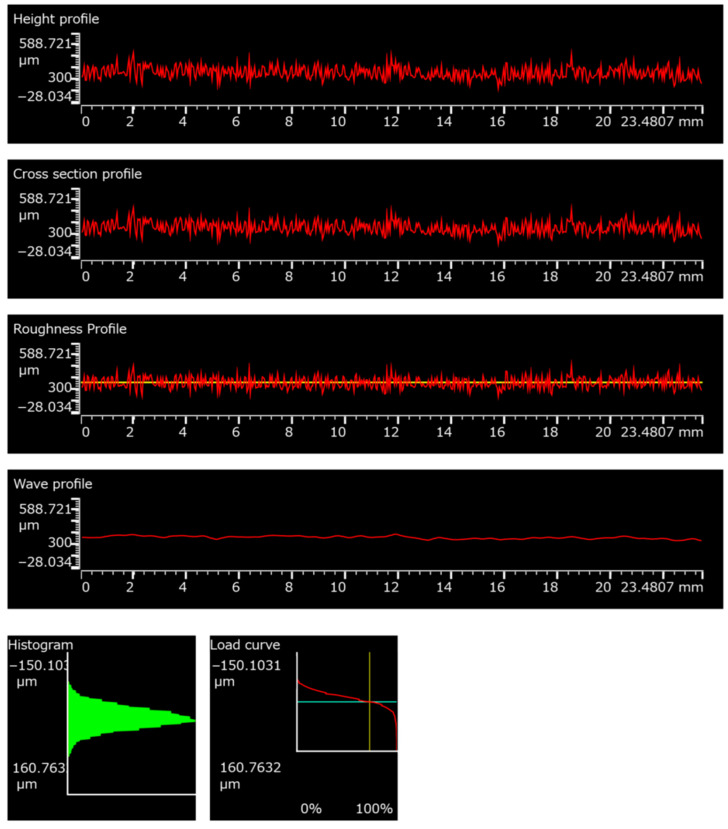
Example of surface roughness measurement of PA12-BC.

The results of surface roughness measurement are shown in Table 2. An example of the roughness measurement result can be seen in Figure 13. The figure presents a comprehensive surface topography analysis consisting of six graphical outputs derived from profilometric measurements over a scan length of approximately 23.48 mm. The vertical axis in all profile graphs is expressed in micrometers [µm].

- Height profile—The height profile represents the raw measured surface topography along the evaluation length. The red curve shows relatively small-amplitude fluctuations around a mean height level, indicating a moderately textured surface without pronounced deep valleys or high peaks. The signal appears statistically stable across the entire scan length.
- Cross-section profile—The cross-section profile corresponds to the leveled surface profile after removal of form or tilt. The overall trend remains similar to the height profile, confirming that no significant macroscopic shape deviation (e.g., curvature) dominates the measurement. Surface irregularities are distributed uniformly along the measured track.
- Roughness profile—The roughness profile isolates the high-frequency components of the surface by filtering out waviness. The red curve oscillates closely around the central reference line (yellow), indicating fine-scale asperities. The amplitude of these fluctuations is relatively low and consistent, suggesting homogeneous micro-roughness.
- Wave profile—The wave (waviness) profile shows the low-frequency surface components after the removal of roughness. The curve is smooth and exhibits only minor long-wavelength undulations, confirming that the surface does not exhibit significant large-scale form deviations.
- Histogram—The histogram illustrates the statistical distribution of surface height values. The distribution appears relatively symmetrical and concentrated around the mean level, indicating a balanced proportion of peaks and valleys with no extreme outliers.
- Load curve (Abbott–Firestone curve)—The load curve represents the cumulative material ratio as a function of profile depth. The S-shaped curve suggests a typical bearing area behavior, with a gradual material transition from peaks to core roughness and valleys. This indicates a surface with moderate load-bearing capacity and relatively uniform texture distribution.

Additional experimental details and supplementary data supporting the presented results are provided in the Supplementary Materials, where the equivalent surface profile analyses (including the height profile, roughness profile, histogram, and Abbott–Firestone curve) are provided for all the surface treatment groups.

The surface roughness analysis revealed significant differences between the individual coating systems.

The manually applied ceramic coating exhibited the highest average Ra values, which can be attributed to the inherent variability of manual layer deposition and local thickness fluctuations. In contrast, the spray-applied coating demonstrated more homogeneous surface morphology, reflected by lower roughness values and reduced statistical dispersion.

The base PA12GB material showed the lowest surface roughness prior to the coating application, confirming that the additive manufacturing process (MJF) produces relatively uniform surface characteristics compared to subsequently applied protective layers.

When comparing coated groups, it is evident that the coating application method plays a more dominant role in final surface morphology than the substrate itself. The increased roughness observed in thicker coating regions suggests that local agglomeration and incomplete leveling during curing may contribute to peak formation, as confirmed by the Abbott–Firestone curve distribution.

Overall, the results indicate that while coating application improves abrasion resistance, it simultaneously modifies the surface topology, potentially affecting tribological behavior. The standard deviation values indicate that the manual application process results in higher variability compared to the controlled spray deposition, confirming the reduced repeatability of manual coating techniques.

ANOVA analysis revealed statistically significant differences in friction coefficients between the investigated surface treatments (*p* < 0.001). Due to the limited measurement count, the roughness statistical evaluation should be interpreted with caution.

### 3.3. Surface Layer Abrasion Test According to ASTM G99-23

**Figure 14:**
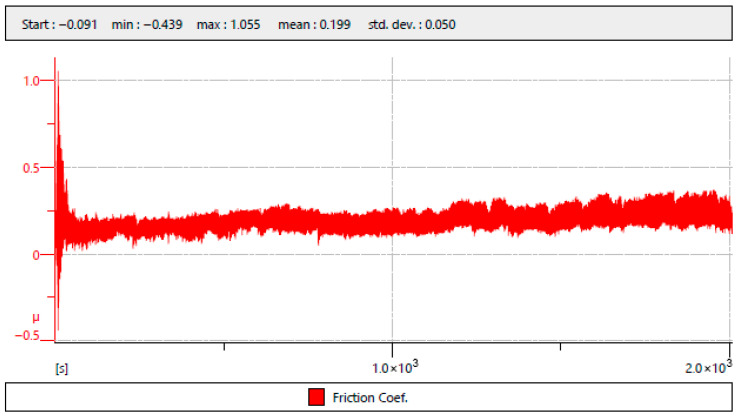
Surface abrasion test measurement example of PA12-AC.

Next, a surface layer wear test was performed in accordance with ASTM G99-23. The test was performed using an Anton Paar TRB3 device (Antot Paar, Prague, Czech Republic). The test parameters were as follows:

- Sample movement: rotational, 300 rpm;
- Rotation radius: 3 mm (distance from the center of rotation of the sample to the center of the friction body);
- Friction body: ball, material 100Cr6 steel, diameter 6 mm;
- Normal load: 10 N;
- Total distance: 188.5 m;
- Number of cycles (rotations): 10,000;
- Sample preparation: cleaning in an ultrasonic cleaner for 5 min in ethanol before testing;

The results of the surface abrasion test measurement are shown in Table 3. An example of the surface abrasion test measurement result can be seen in Figure 14. The figure shows the evolution of the friction coefficient (μ) as a function of time during a tribological test. The horizontal axis represents time in seconds [s], extending to approximately 2000 s, while the vertical axis represents the friction coefficient μ.

At the beginning of the test, a pronounced transient peak is observed, with the friction coefficient reaching a maximum value of approximately 1.055 and a minimum of −0.439. After this initial running-in phase, the signal stabilizes and fluctuates around a relatively steady level. Over the majority of the test’s duration, the friction coefficient gradually increases, showing small oscillations typical of sliding contact under constant load.

The statistical parameters displayed at the top of the figure indicate a mean friction coefficient of 0.199 with a standard deviation of 0.050. The overall trend suggests an initial stabilization period followed by a slightly increasing friction behavior, likely associated with progressive surface adaptation or mild wear during the sliding test.

The test results show that a comparable coefficient of friction to the original condition without surface treatment (f = 0.1312) was only achieved with PostPro3D vaporization surface treatment (f = 0.1251) and AC surface treatment (f = 0.1375). In samples with glasscoat surface treatment (f = 0.4133) and samples with ceramic coating (f = 0.4790), there was a significant increase in the coefficient of friction, approximately 3–4 times higher than in the initial state without surface treatment. In contrast, samples with a BC surface treatment showed a significant reduction in the coefficient of friction (f = −0.0071) to a value that can be considered almost zero.

ANOVA analysis revealed statistically significant differences in friction coefficients between the investigated surface treatments (*p* < 0.001).

### 3.4. Measuring the Hardness of Surface Layers

The hardness of the surface layers was also measured and the hardness of individual surface treatments was determined according to ASTM D2240-15R21—Shore D. The measurement was performed using a SAUTER HBD 100 durometer (Sauter Metall GmbH, Prague, Czech Republic).

This durometer is suitable for measuring materials such as hard rubber, hard synthetic materials, thermoplastics, vinyl sheets, cellulose acetates, and MDF. The penetration depth is 0–2.5 mm, and the measurement range is 0–100 Shore.

The results of the surface layer hardness measurement are shown in Table 4.

The measurement results show that the BC (75 Shore D) and PostPro3D vaporization (76 Shore D) surface treatments exhibit almost identical surface roughness compared to the initial state without surface treatment (75 Shore D). In contrast, an increase in surface hardness was observed in the case of AC (78 Shore D), glasscoat (84 Shore D), and ceramic spray (84 Shore D) surface coatings.

Statistical analysis confirmed significant differences in hardness between the evaluated coating systems (*p* < 0.001).

### 3.5. Correlation Between Surface Roughness and Friction Coefficient

To evaluate the influence of surface morphology on tribological behavior, a regression analysis was performed between the average surface roughness (Ra) and the measured coefficient of friction (COF). The results indicate a positive correlation between increasing roughness and the friction coefficient. The obtained linear regression model suggests that surface roughness contributes significantly to the observed friction behavior of the coating systems.

A linear regression analysis was performed to evaluate the relationship between surface roughness (Ra) and the coefficient of friction (COF). The resulting regression model can be expressed as:

COF = 0.0053Ra + 0.1411(1)

**Figure 15:**
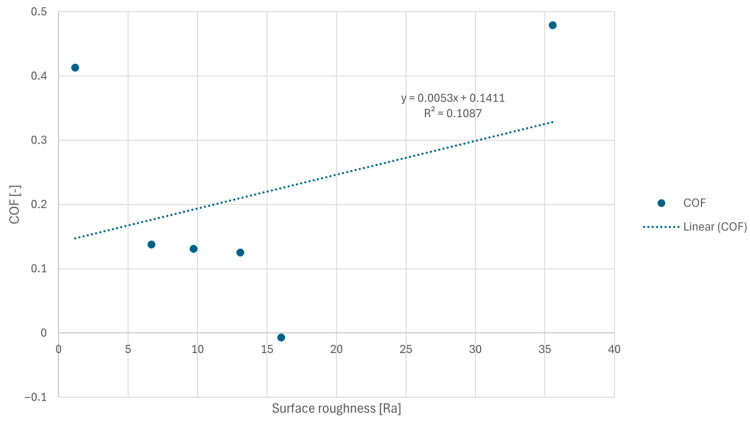
Linear regression between average surface roughness (Ra) and coefficient of friction (COF) for the investigated coating systems.

The positive slope of the regression line indicates that an increase in surface roughness tends to increase the friction coefficient. However, the relatively low coefficient of determination (R^2^ = 0.1087) suggests that surface roughness alone explains only a limited portion of the variability in friction behavior, as can be seen in Figure 15.

This indicates that additional factors, such as coating composition, hardness, and surface morphology, also significantly influence the tribological response of the investigated systems.

It should also be noted that the regression model is based on the averaged values of five coating systems and therefore represents an indicative relationship rather than a fully predictive model.

## 4. Discussion

The results obtained in this study are consistent with previous research investigating the surface modification of additively manufactured polyamide components. Several studies have reported that protective coatings and surface post-processing techniques can significantly improve the wear resistance and tribological performance of polymer-based AM parts.

In particular, ceramic-reinforced coatings have been shown to enhance surface hardness and reduce abrasive wear due to the load-bearing effect of hard particles dispersed in the matrix. Similarly, solvent vapor smoothing techniques have been reported to reduce surface roughness and modify the near-surface morphology of polyamide materials.

The findings of the present work therefore align with the current academic understanding of surface modification strategies for additively manufactured polymers, while also providing a comparative evaluation of several commercially available coating systems.

The experimental results clearly demonstrate that the selected surface treatments significantly modify the surface characteristics of MJF-produced PA12GB components. The observed differences can be interpreted in terms of layer thickness, surface morphology, and intrinsic properties of the applied coatings.

The vapor smoothing process (PostPro3D) produced the thinnest modified layer and maintained surface properties close to the untreated reference state in terms of hardness and friction coefficient. This indicates that chemical vapor smoothing primarily modifies surface topography rather than introducing a mechanically distinct coating layer. Although roughness slightly increased compared to the untreated sample, the friction coefficient remained comparable, suggesting that smoothing may homogenize asperities without fundamentally altering tribological interaction.

Base coating (BC) resulted in a moderate layer thickness but showed an anomalous near-zero average friction coefficient. This unexpected behavior may indicate measurement artifacts, unstable contact conditions, or specific interaction between the coating and the steel counter-body. Further investigation would be necessary to confirm the reproducibility of this result.

Acrylic coating (AC) significantly reduced surface roughness while moderately increasing hardness. However, the coefficient of friction remained comparable to the reference material. This suggests that smoother surface morphology alone does not necessarily lead to lower friction under the tested contact conditions.

Glasscoat and ceramic coatings formed substantially thicker and harder layers. Both treatments markedly increased surface hardness (≈84 Shore D) but also led to a 3–4-fold increase in friction coefficient. This behavior is consistent with the formation of hard, possibly brittle surface layers that increase the adhesion and plowing components of friction when sliding against a steel ball.

From a functional perspective, the results indicate that no single surface treatment universally improves all performance parameters. Treatments increasing hardness may simultaneously increase friction. Therefore, the selection of a suitable surface treatment must be application-specific. Future research should include wear volume quantification, temperature-dependent tribological testing, adhesion strength evaluation of coatings, and long-term durability analysis under cyclic loading.

From a composite materials perspective, the presence of glass microspheres in PA12GB may significantly influence coating adhesion mechanisms. The rigid filler particles increase the effective surface stiffness and introduce local heterogeneity at the coating–substrate interface. This may alter stress distribution during sliding contact and potentially promote micro-debonding at regions with mismatched elastic properties. Furthermore, the partial exposure of glass beads at the surface could modify wetting behavior and limit chemical compatibility between the coating matrix and the polyamide phase. Since neat PA12 was not included in the present study, the isolated effect of glass reinforcement on interfacial bonding cannot be quantitatively assessed. This represents a limitation of the current work and will be addressed in future investigations.

The relatively high thickness deviation observed for the ceramic-filled epoxy coating is attributed to manual application. Variations in layer thickness may influence local contact mechanics and friction behavior. Although the overall trend of increased hardness and friction remains clear, improved deposition control (e.g., automated spraying or controlled film applicators) would enhance reproducibility in future investigations.

Although tribological performance provides indirect information about coating stability, direct adhesion strength measurements were not included in this study. Standardized cross-cut or pull-off tests would enable the quantitative evaluation of interfacial bonding strength and failure mechanisms. Since coating durability is fundamentally governed by adhesion properties, future investigations will incorporate adhesion testing to establish a direct correlation between interfacial strength and friction behavior.

The absence of coating delamination or catastrophic failure during 10,000 sliding cycles suggests that, under the applied loading conditions (10 N, 188.5 m sliding distance), interfacial adhesion was sufficient to maintain coating integrity. However, this functional stability does not substitute standardized adhesion strength characterization.

The thickness measurements were performed on one representative specimen per treatment group due to the destructive nature of cross-sectional analysis. Although fifteen local measurements were taken to capture spatial variability, evaluation on a single specimen limits statistical generalization. Future studies should include the cross-sectional analysis of multiple specimens per group to improve statistical robustness and enable formal variance analysis.

The very low *p*-values obtained from the ANOVA analysis confirm that the observed differences between coating systems are not caused by random experimental variability but are primarily driven by the coating type and application method.

The tribological behavior of the investigated surfaces can be interpreted using classical asperity contact theory. Due to surface roughness, the real contact area between two surfaces is significantly smaller than the nominal contact area and is formed primarily by asperity peaks. Increased roughness therefore modifies the distribution of local contact stresses and affects friction behavior. The observed correlation between surface roughness and COF is consistent with this theoretical framework.

The PostPro3D vapor smoothing treatment modifies the surface morphology through controlled polymer surface dissolution and re-solidification. This process may promote the partial reorganization of polymer chains near the surface, which can influence the local crystallinity of the material. Changes in crystallinity can affect the mechanical and tribological properties of the treated surface. Although crystallinity was not directly measured in this study, similar effects have been reported in previous investigations of solvent vapor treatment of polyamide materials.

The superior performance of the ceramic-filled coating can be explained by the presence of hard ceramic particles dispersed within the polymer matrix. These particles act as load-bearing elements and increase the overall hardness and wear resistance of the coating. During sliding contact, the ceramic particles limit the plastic deformation of the polymer matrix and reduce the penetration depth of asperities on the the counterface. As a result, the coating exhibits improved abrasion resistance and more stable tribological behavior compared to purely polymeric surface treatments. Additionally, the ceramic particles contribute to the stabilization of the contact interface by distributing local contact stresses and preventing excessive localized deformation.

The applied surface treatments consist of commercially available coating systems and post-processing technologies with proprietary chemical compositions. Therefore, detailed chemical characterization of the coating layers (e.g., FTIR analysis) was not performed in this study. The primary objective of this work was to evaluate the functional performance of these surface treatments in terms of surface morphology, hardness, and tribological behavior rather than their chemical composition.

The observed differences in tribological behavior can be attributed to variations in coating composition, surface morphology, and mechanical properties of the applied surface layers, which collectively influence the real contact area and wear mechanisms during sliding.

Surface wettability may also influence interfacial interactions; however, the present study primarily focuses on the mechanical and tribological performance of the coatings.

### Limitations of the Study

Despite the promising results obtained in this study, several limitations should be acknowledged. The ceramic-filled coating was applied manually, which may introduce variability in coating thickness and surface morphology. Although the statistical analysis indicated relatively acceptable thickness uniformity, manual application may limit repeatability in large-scale industrial production where automated coating processes would be preferable. Automated coating techniques such as spray deposition or robotic application could improve process consistency.

Furthermore, the experiments were conducted on relatively simple specimen geometries. In practical additive manufacturing applications, components often contain complex geometries such as lattice structures, internal channels, or thin-walled features. The behavior of coating systems on such geometries may differ due to variations in coating accessibility, thickness distribution, and local stress conditions. Therefore, further research should investigate the applicability of the proposed surface treatments on more complex AM structures.

## 5. Conclusions

This study evaluated the influence of several surface treatment methods on the surface properties and tribological behavior of PA12GB components manufactured by Multi-Jet Fusion technology. The investigated treatments included commercially available coating systems as well as a vapor smoothing process (PostPro3D), which were assessed in terms of coating thickness, surface roughness, hardness, and coefficient of friction.

The results demonstrated that the applied surface treatments significantly modified the surface characteristics of the printed components. Statistical analysis confirmed that the type of surface treatment had a statistically significant influence on coating thickness, hardness, surface roughness, and friction behavior. At the same time, regression analysis between surface roughness and the coefficient of friction revealed only a weak correlation, indicating that tribological performance is governed not only by surface morphology but also by additional factors such as coating composition and mechanical properties.

Among the evaluated surface treatments, the ceramic-filled coating system showed the most favorable tribological performance, providing improved abrasion resistance and increased surface hardness compared to untreated and alternatively treated specimens. The presence of ceramic particles in the coating layer likely enhances the load-bearing capacity of the surface and increases resistance to mechanical wear at the contact interface.

The presented results provide a systematic comparative evaluation of selected industrial surface treatment technologies applied to additively manufactured polymer components. The findings contribute to a better understanding of how post-processing techniques influence the functional performance of MJF-produced parts and may support the selection of suitable surface finishing strategies for applications where improved tribological properties are required.

Future work may focus on a deeper investigation of the interaction between coating composition, surface microstructure, and long-term wear behavior, particularly for components with more complex geometries typical of additive manufacturing.

## Figures and Tables

**Table 1:** Results of surface layer thickness measurements.

Layer Thickness [μm]					
Measurement No.	PA12-BC	PA12-PostPro3d	PA12-AC	PA12-Glasscoat	PA12-Ceramic
1	98.7	48.4	82.5	270.6	462.9
2	90.7	45.4	107.7	263.2	383.0
3	116.7	59.5	130.3	232.7	436.5
4	96.9	52.8	144.8	259.6	418.9
5	83.0	53.6	81.5	269.9	411.6
6	106.5	49.8	104.3	252.2	398.4
7	78.6	58.3	86.8	287.5	413.0
8	96.7	47.6	127.4	296.3	421.8
9	78.3	41.6	101.4	253.3	404.2
10	90.0	27.1	68.4	253.7	427.7
11	98.2	54.4	89.0	270.6	383.7
12	92.8	35.5	89.1	245.2	400.6
13	86.5	28.1	153.3	238.2	358.0
14	86.6	38.0	129.4	293.0	433.6
15	96.7	28.1	100.4	284.8	383.0
Mean Average	93.1	44.5	106.4	264.7	409.1
Standard Deviation [-]	10.2	11.0	25.2	19.6	26.3
Variance [-]	104.8	120.8	636.0	383.0	692.8
ANOVA *p*-value [-]	<0.001				

**Table 2:** Results of surface roughness measurements for individual surface finishes.

	Ra (According to ISO 21920-2:2021)					
Measurement No.						
Sample	1	2	Mean Average	Standard Deviation [-]	Variance [-]	ANOVA *p*-Value [-]
PA12-without ST	9.272	10.127	9.700	0.605	0.366	<0.001
PA12-BC	17.042	15.011	16.027	1.436	2.062	
PA12-postpro3D	12.438	13.702	13.070	0.894	0.799	
PA12-AC	6.091	7.276	6.684	0.838	0.702	
PA12-glasscoat	1.095	1.269	1.182	0.123	0.015	
PA12-ceramic	34.475	36.671	35.573	1.553	2.411	

**Table 3:** Results of surface layer abrasion test measurements.

Average Coefficient of Friction During the Test							
Measurement No.							
Sample	1	2	3	Mean Average	Standard Deviation [-]	Variance [-]	ANOVA *p*-Value [-]
PA12-without ST	0.1289	0.1275	0.1372	0.1312	0.0052	0.0000	<0.001
PA12-BC	−0.0023	−0.0071	−0.0120	−0.0071	0.0049	0.0000	
PA12-PostPro3D	0.1269	0.1287	0.1197	0.1251	0.0048	0.0000	
PA12-AC	0.1705	0.0433	0.1986	0.1375	0.0828	0.0068	
PA12-glasscoat	0.4024	0.5182	0.3192	0.4133	0.0999	0.0100	
PA12-ceramic	0.5254	0.4540	0.4575	0.4790	0.0403	0.0016	

**Table 4:** Results of surface finish hardness measurements.

	Hardness Shore D									
Measurement No.										
Sample	1	2	3	4	5	6	Mean Average	Standard Deviation [-]	Variance [-]	ANOVA *p*-Value [-]
PA12-without ST	75	75	74	75	77	75	75	1	1	<0.001
PA12-BC	73	76	74	75	76	77	75	1	2	
PA12-PostPro3D	75	76	75	76	75	76	76	1	0	
PA12-AC	78	78	78	80	77	78	78	1	1	
PA12-glasscoat	82	84	84	85	83	84	84	1	1	
PA12-ceramic	85	85	84	83	84	85	84	1	1	

## Data Availability

The original contributions presented in this study are included in the article/Supplementary Materials. Further inquiries can be directed to the corresponding author.

## Supplementary Materials

The following supporting information can be downloaded at: https://www.mdpi.com/article/10.3390/polym18060703/s1. Figure S1: Surface roughness measurement of PA12-without ST; Figure S2: Surface roughness measurement of PA12-BC; Figure S3: Surface roughness measurement of PA12-PostPro3D; Figure S4: Surface roughness measurement of PA12-AC; Figure S5: Surface roughness measurement of PA12-glasscoat; Figure S6: Surface roughness measurement of PA12-ceramic.

## Abbreviations

The following abbreviations are used in this manuscript:

AM	Additive manufacturing
ST	Surface treatment
PA12	Polyamide 12, known as nylon
PA12GB	Glass bead-filled PA12
MJF	Multi-Jet Fusion
FDM	Fused Deposition Modeling
SLS	Selective Laser Sintering
BC	Base coating
AC	Acrylic coating
AMT	Additive Manufacturing Technologies
COF	Coefficient of friction
ANOVA	Analysis of Variance
SD	Standard deviation
ISO	International Organization for Standardization
ASTM	American Society for Testing and Materials
UV	Ultraviolet

## References

[1] Lee P.-H., Chung H., Lee S.W., Yoo J., Ko J. Review: Dimensional Accuracy in Additive Manufacturing Processes. Proceedings of the ASME 2014 International Manufacturing Science and Engineering Conference.

[2] Kechagias J., Chaidas D., Vidakis N., Salonitis K., Vaxevanidis N.M. (2022). Key Parameters Controlling Surface Quality and Dimensional Accuracy: A Critical Review of FFF Process. Mater. Manuf. Process..

[3] Shim J.S., Kim J.-E., Jeong S.H., Choi Y.J., Ryu J.J. (2020). Printing accuracy, mechanical properties, surface characteristics, and microbial adhesion of 3D-printed resins with various printing orientations. J. Prosthet. Dent..

[4] Ogazi A.C., Otunniyi I.O., Mauchline D.A. (2025). Production of PA12 Powder for Additive Manufacturing: Progress, Challenges, and Prospects. Polym. Adv. Technol..

[5] 3DPrint.com Company HP Reveals More Info About Their Multi Jet Fusion 3D Printing Technology, Plans for Second 3D Printer, 2016. https://3dprint.com/113630/hp-multi-jet-fusion-plans-info/.

[6] Avanzini A., Battini D., Pandini S. (2022). Static and fatigue behavior in presence of notches for polyamide 12 (pa12) additively man-ufactured via multi jet fusion™ process. Int. J. Fatigue.

[7] O’Connor H.J., Dowling D.P. (2020). Comparison between the properties of polyamide 12 and glass bead filled polyamide 12 using the multi jet fusion printing process. Addit. Manuf..

[8] De Coninck H., Meyers S., Van Puyvelde P., Van Hooreweder B. (2024). On the Difference in Mechanical Behavior of Glass Bead-Filled Polyamide 12 Specimens Produced by Laser Sintering and Injection Molding. 3D Print. Addit. Manuf..

[9] Rohrsen N.C., Hagedorn D. (2024). Improving the surface quality of additive manufactured polyamide parts using conventional treatment methods. Int. J. Adv. Manuf. Technol..

[10] Turek P., Bazan A., Zakrecki A. (2023). Influence of post-processing treatment on the surface roughness of polyamide PA12 samples manufactured using additive methods in the context of the production of orthoses. Proc. Inst. Mech. Eng. Part B J. Eng. Manuf..

[11] Hassanpour M., Narongdej P., Alterman N., Moghtadernejad S., Barjasteh E. (2024). Effects of Post-Processing Parameters on 3D-Printed Dental Appliances: A Review. Polymers.

[12] Holländer A., Cosemans P. (2020). Surface technology for additive manufacturing. Plasma Process. Polym..

[13] Zentgraf J., Nützel F., Mühlbauer N., Schultheiss U., Grad M., Schratzenstaller T. (2024). Surface Treatment of Additively Manufactured Polyetheretherketone (PEEK) by Centrifugal Disc Finishing Process: Identification of the Key Parameters. Polymers.

[14] Kariž M., Tomec D.K., Dahle S., Kuzman M.K., Šernek M., Žigon J. (2021). Effect of Sanding and Plasma Treatment of 3D-Printed Parts on Bonding to Wood with PVAc Adhesive. Polymers.

[15] Suárez-Macías J., Terrones-Saeta J.M., Iglesias-Godino F.J., Corpas-Iglesias F.A. (2020). Surface Treatments with Dichloromethane to Eliminate Printing Lines on Polycarbonate Components Printed by Fused Deposition Modelling Technology. Materials.

[16] Tamașag I., Beșliu-Băncescu I., Severin T.-L., Dulucheanu C., Cerlincă D.-A. (2023). Experimental Study of In-Process Heat Treatment on the Mechanical Properties of 3D Printed Thermoplastic Polymer PLA. Polymers.

[17] Jovanović J., Đukanović M., Radunović L., Vuković S.R., Jovanović M. (2025). Enhanced Mechanical Properties and Surface Finish of PLA 3D Prints via Combined Heat Annealing and Powder Coating. Appl. Sci..

[18] Kostadinov G., Penyashki T., Nikolov A., Vencl A. (2024). Improving the Surface Quality and Tribological Characteristics of 3D-Printed Titanium Parts through Reactive Electro-Spark Deposition. Materials.

[19] Myalski J., Godzierz M., Olesik P. (2020). Effect of Carbon Fillers on the Wear Resistance of PA6 Thermoplastic Composites. Polymers.

[20] Mittal K.L. (2003). Adhesion Aspects of Polymeric Coatings.

[21] Kinloch A. (1987). Adhesion and Adhesives: Science and Technology.

[22] Károly Z., Kalácska G., Sukumaran J., Fauconnier D., Kalácska Á., Mohai M., Klébert S. (2019). Effect of Atmospheric Cold Plasma Treatment on the Adhesion and Tribological Properties of Polyamide 66 and Poly(Tetrafluoroethylene). Materials.

[23] Mashayekhi F., Bardon J., Westermann S., Addiego F. (2021). Adhesion Optimization between Incompatible Polymers through Interfacial Engineering. Polymers.

[24] (2021). Geometrical Product Specifications (GPS)—Surface Texture: Profile—Part 2: Terms, Definitions and Surface Texture Parameters.

[25] (2023). Standard Test Method for Wear Testing with a Pin-on-Disk Apparatus.

[26] (2021). Standard Test Method for Rubber Property—Durometer Hardness.

[27] Sezemský J., Primc G., Vacková T., Jeníková Z., Mozetič M., Špatenka P. (2025). Enhanced Mechanical Properties of 3D-Printed Glass Fibre-Reinforced Polyethylene Composites. Polymers.

[28] Bazan A., Turek P., Zakręcki A. (2023). Influence of Antibacterial Coating and Mechanical and Chemical Treatment on the Surface Properties of PA12 Parts Manufactured with SLS and MJF Techniques in the Context of Medical Applications. Materials.

[29] Balan G.S., Raj S.A., Adithya R.N. (2024). Effect of post-heat treatment on the mechanical and surface properties of nylon 12 produced via material extrusion and selective laser sintering processes. Polym. Bull..

[30] Muna I.I., Mieloszyk M., Rimasauskiene R., Maqsood N., Rimasauskas M. (2022). Thermal Effects on Mechanical Strength of Additive Manufactured CFRP Composites at Stable and Cyclic Temperature. Polymers.

[31] Bahrami M., Abenojar J., Martínez M.A. (2021). Comparative Characterization of Hot-Pressed Polyamide 11 and 12: Mechanical, Thermal and Durability Properties. Polymers.

[32] Raz K., Chval Z., Hula F., Markopoulos A. (2025). Surface Treatment and Analysis of 3D-Printed Plastic Molds for Prototype and Small-Series Injection Molding. Polymers.

[33] Khosravani M.R., Schüürmann J., Berto F., Reinicke T. (2021). On the Post-Processing of 3D-Printed ABS Parts. Polymers.

[34] Bolat Ç., Demircan F., Gür İ., Yalçın B., Şener R., Ercetin A. (2025). Hardness and Surface Roughness of 3D-Printed ASA Components Subjected to Acetone Vapor Treatment and Different Production Variables: A Multi-Estimation Work via Machine Learning and Deep Learning. Polymers.

[35] Chun S.-Y., Lee G., Kim S.-j., Jeong B., Shin J., Cho I., Kim H.-D., Lee H., Kim T. (2021). Effects of Post-Treatment to Improve the Surface Quality of 3D Printing Cement Mold Casting. Appl. Sci..

[36] Bourgi R., Etienne O., Holiel A.A., Cuevas-Suárez C.E., Hardan L., Roman T., Flores-Ledesma A., Qaddomi M., Haikel Y., Kharouf N. (2025). Effectiveness of Surface Treatments on the Bond Strength to 3D-Printed Resins: A Systematic Review and Meta-Analysis. Prosthesis.

[37] (2020). Paints and varnishes—Cross-cut test.

[38] (2023). Paints and varnishes—Pull-off test for adhesion.

