# Supplementary material for: Feasibility Study of Suitable Surface Treatments for 3D-Printed Parts to Increase Abrasion Resistance Stability
Source: Polymers (Basel). 2026 Mar 13;18(6):703. doi: 10.3390/polym18060703 (PMC13029864; doi:10.3390/polym18060703)
Supplement: Supplementary file 1 [file polymers-18-00703-s001.zip › polymers-4193618-supplementary.pdf]

Supplementary Material

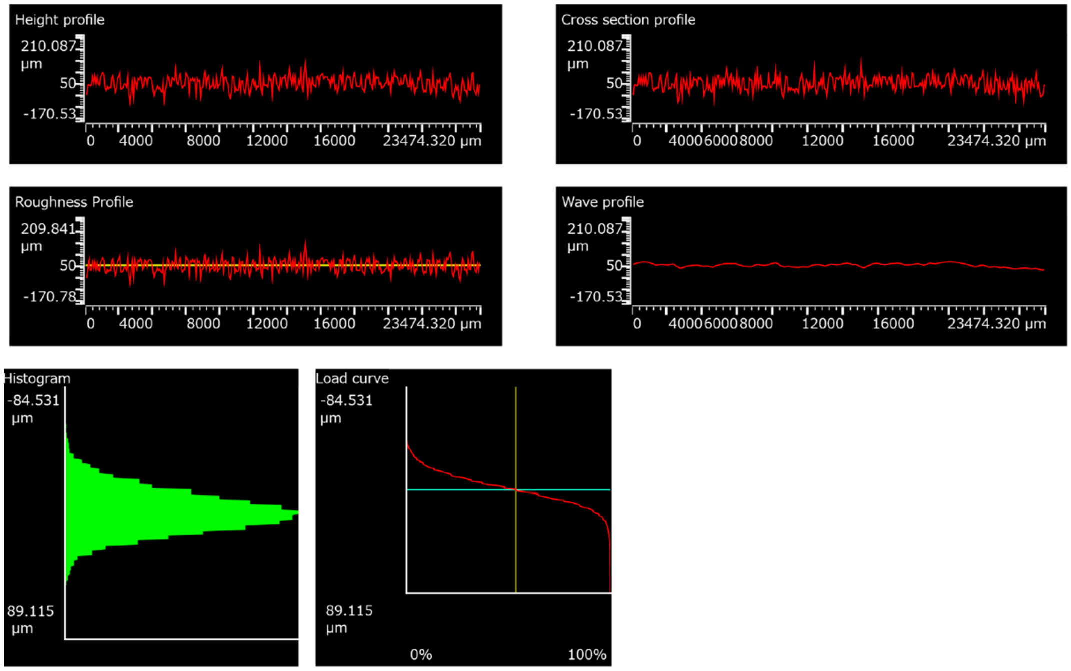

Figure S1. Surface roughness measurement of PA12-without ST.

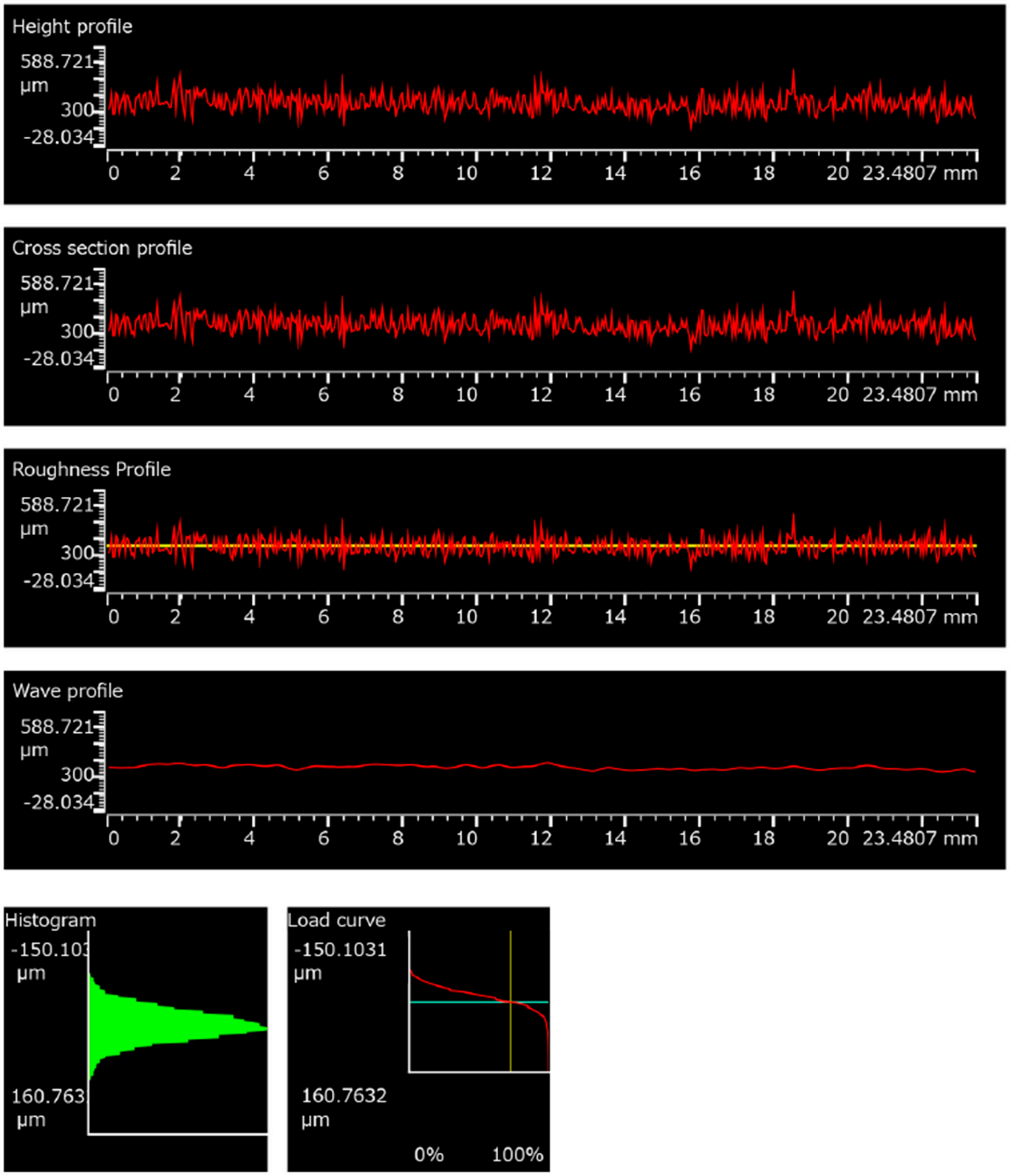

Figure S2. Surface roughness measurement of PA12-BC.

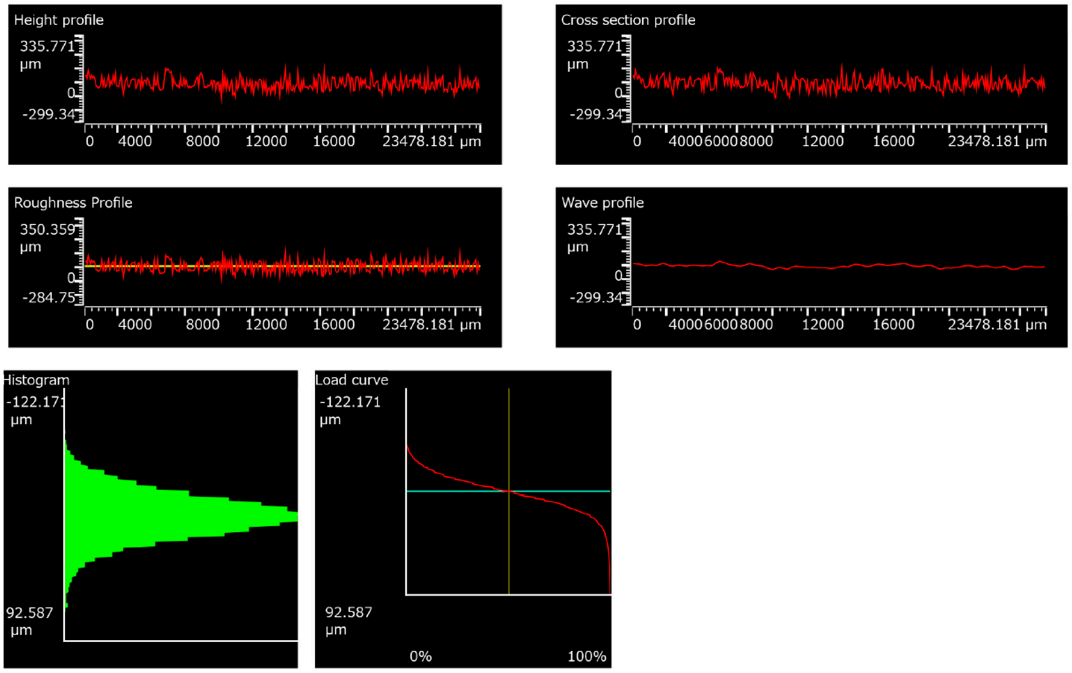

Figure S3. Surface roughness measurement of PA12-PostPro3D.

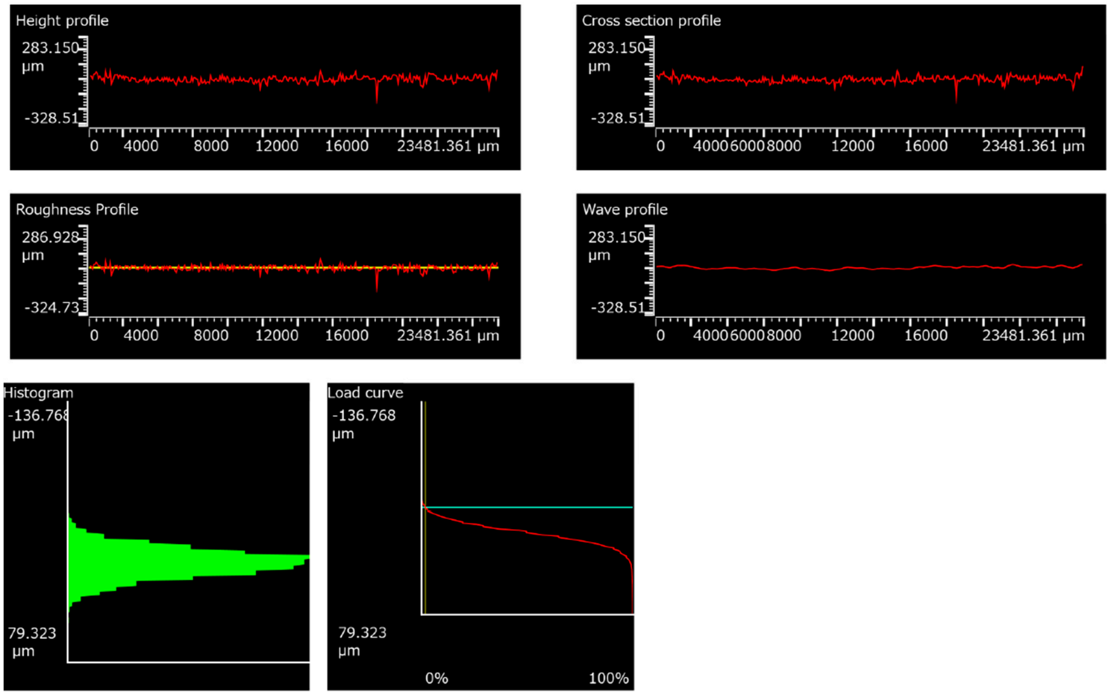

Figure S4. Surface roughness measurement of PA12-AC.

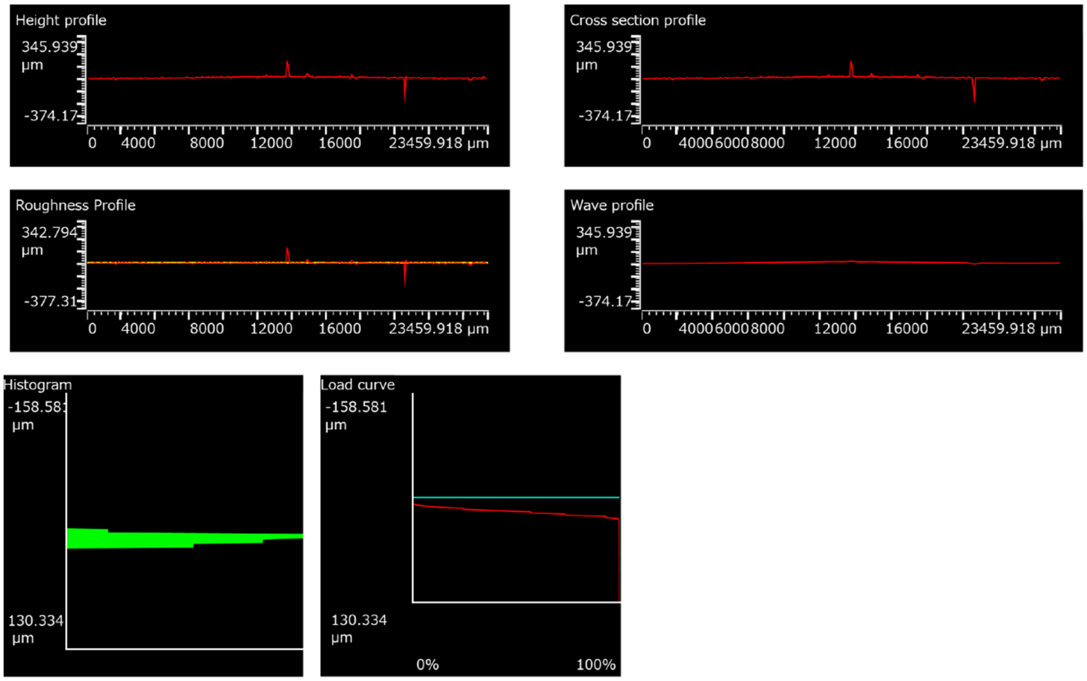

Figure S5. Surface roughness measurement of PA12-glasscoat.

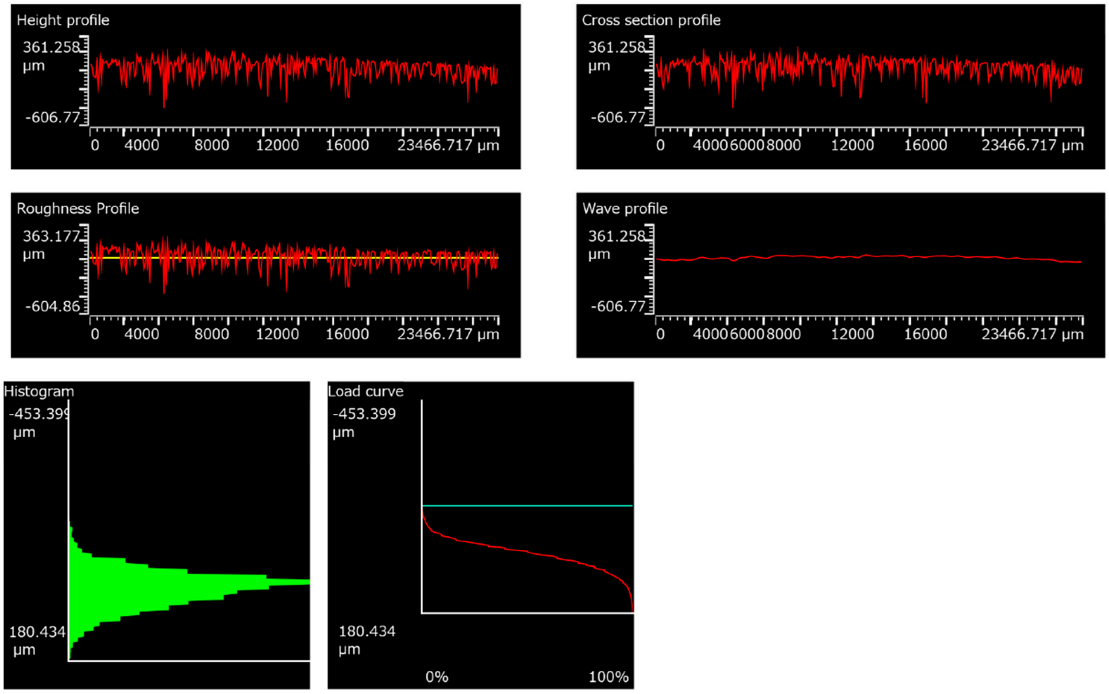

Figure S6. Surface roughness measurement of PA12-ceramic.
